# Customized SmartPeg for measurement of resonance frequency of mini dental implants

**DOI:** 10.1186/s40729-017-0066-6

**Published:** 2017-02-01

**Authors:** Jagjit Singh Dhaliwal, Rubens F. Albuquerque, Ali Fakhry, Sukhbir Kaur, Jocelyne S. Feine

**Affiliations:** 1 Faculty of Dentistry, McGill University, 2001 McGill College Avenue, Suite 500, Montreal, Quebec, H3A 1G1 Canada; 2 Faculty of Dentistry of Ribeirão Preto, University of São Paulo, Ribeirão Preto, SP Brazil; 3 Department of Zoology, Panjab University, Chandigarh, India; 4 PAPRSB Institute of Health Sciences, Universiti Brunei Darussalam, ᅟ, Brunei Darussalam

## Abstract

**Background:**

One-piece narrow diameter implants (NDIs) have been recommended as “Single-tooth replacements in the anterior zones, single posterior, multiple-unit fixed dental prosthesis (FDP), edentulous jaws to be rehabilitated with FDP, and edentulous jaws rehabilitation with overdentures in situations with reduced mesiodistal space or reduced ridge width.” (ITI consensus 2013). Since NDIs can be immediately loaded, it is important to be able to carry out stability testing. We developed and validated a customized SmartPeg for this type of implant to measure the Implant Stability Quotient (ISQ). The ISQ of mini dental implants (MDIs) was measured and compared with the stability of standard and in a rabbit model.

**Objective:**

The aim of the study is to test the feasibility of a customized SmartPeg for resonance frequency measurement of single-piece mini dental implants and to compare primary stability of a standard and the mini dental implant (3M™ESPE™ MDI) in a rabbit model after 6 weeks of healing.

**Methods:**

Eight New Zealand white rabbits were used for the study. The protocol was approved by the McGill University Animal Ethics Review Board. Sixteen 3M™ESPE™ MDI and equal number of standard implants (Ankylos® Friadent, Dentsply) were inserted into the tibia/femur of the rabbits and compared. Each rabbit randomly received two 3M™ESPE™ MDI and two Ankylos® implants in each leg. ISQ values were measured with the help of an Osstell ISQ device using custom-made SmartPegs for the MDIs and implant-specific SmartPegs™ (Osstell) for the Ankylos®. Measurements were obtained both immediately following implant placement surgery and after a 6-week healing period. Each reading was taken thrice and their average compared using Wilcoxon matched pairs signed-rank tests.

**Results:**

The median ISQ and interquartile range (IQR) values were 53.3 (8.3) at insertion and 60.5 (5.5) at 6 weeks for the 3M™ESPE™MDI and, respectively, 58.5 (4.75) and 65.5 (9.3) for the Ankylos® implant. These values also indicate that both types of implants achieved primary and secondary stability, and this is supported by histological data. ISQ values of both 3M™ESPE™ MDI and Ankylos® increased significantly from the time of insertion to 6 weeks post-insertion (*p* < 0.05).

**Conclusions:**

The new custom-made SmartPeg is suitable for measuring the Implant Stability Quotient of 3M™ESPE™MDIs. The primary stability of 3M™ESPE™MDIs is similar to the primary stability attained by standard implants in the rabbit tibia.

## Background

Osseointegration refers to the phenomenon for close apposition of the bone to the surface of an implant with no interposing tissue that can be clinically demonstrated by absence of mobility [1, 2]. Obtaining primary stability seems to be a precondition for a successful osseointegration [3]. Dental implants have a success rate of over 90% and are available in various sizes with different surfaces [4, 5]. The diameter of dental implants usually ranges from 3 mm (narrow diameter) to 7 mm (wide diameter), with the majority falling in the “standard diameter” range of 3.7 to 4.0 mm.

Single-piece mini dental implants (MDIs) or narrow diameter implants (NDIs) are being widely used for stabilizing complete dentures [6], orthodontic anchorage [7, 8], single-tooth replacements, and fixing surgical guides for definitive implant placement, and as transitional implants for support of interim removable prosthesis during the healing phase of final fixtures [9–11].

Due to the MDIs’ narrower diameter (1.8–2.4 mm) as compared with regular implants, the width of the bone required for their placement is smaller, making the surgery minimally invasive as compared with the surgery for conventional implant insertion [12]. In addition, transmucosal placement is performed using a single pilot drill, reducing the need for sutures and long recovery periods [13]. Mini dental implants can also be immediately loaded and are cost-effective, which makes them an advantageous alternative for mandibular implant overdentures [13, 14]. The success of these implants will depend, however, on their capacity to outstand functional loadings.

Osseointegrated implants are clinically characterized by the absence of mobility, which can be assessed by measuring the primary and secondary implant stability [15, 16]. Some authors have suggested that primary stability is a critical factor in predicting whether an implant will be successful or not, and it is considered of highest importance in the long-term success of dental implants [17, 18]. It has also been reported that micro movements can be detected at an early stage by measuring the primary implant stability and that they are unfavorable to the osseointegration of dental implants [19–21].

Mechanical testing methods like reverse torque, or “pullout test,” have been used to study and measure the mechanical interface between implant and bone in various ways [22, 23]. The Branemark group has evaluated the mechanical properties of osseointegrated implants using torsion and pullout tests and lateral loading tests [24, 25]. Presence or absence of mobility and the bone level around the implant can be estimated by non-invasive methods based on resonance frequency analysis (RFA) such as those used by Periotest and Osstell™ devices [26–30].

Resonance frequency analysis has been used to document changes in the bone healing along the implant-bone interface by measuring the stiffness of implant in the bone tissue [31–34]. It has also been used to determine whether implants are ready for the final restoration [35] or ready to be loaded [33] and to identify the implants at “risk” [36]. The first studies using RFA were published in 1996 [37]. In 1997, Meredith et al. suggested a non-invasive method for determining the resonance frequency associated with dental implants by connecting an adapter/transducer onto the abutment in an animal study [38]. The experimented RFA system, base on magnetic pulses, has been commercially produced as Osstell since the year 2000 [19] (Osstell AB, Göteborg, Sweden). Osstell was later followed by Osstell Mentor™ and Osstell ISQ™. It calculates the Implant Stability Quotient (ISQ) converting kilohertz units to ISQ on a scale of 1–100, where 100 signifies the highest implant stability. Increases in ISQ measurements indicate improved bone stiffness and healing around the implant and better implant stability. The Osstell ISQ works by introducing a controlled vibration to the implant by means of a sensor and a rod (SmartPeg) connected to the implant and measuring its frequency. These SmartPegs are usually fabricated for standard diameter implants. The osseointegration potential of single-piece mini dental implants (3M™ESPE™ MDIs) has never been assessed by RFA. The immediate post-surgical ISQ assessment of MDIs is particularly relevant due to their smaller size and surface area in comparison to standard implants.

There are no published studies on the ISQ measurement of mini dental implants, as SmartPegs for these implants are not available till date. Since these are one-piece implants and do not have an internal thread for the SmartPeg’s attachment, a custom-made SmartPeg needs to be fabricated for ISQ measurement. Therefore, we developed and tested a customized SmartPeg for 3M™ESPE™ MDIs to measure the ISQ.

## Objective

The aim of the study is to test the feasibility of a customized SmartPeg for ISQ measurement of single-piece mini dental implants and to compare the primary stability of a standard and the mini dental implant (3M™ESPE™MDI) in a rabbit model after 6 weeks of healing.

## Methods

### Development of a customized SmartPeg

**Fig. 1:**
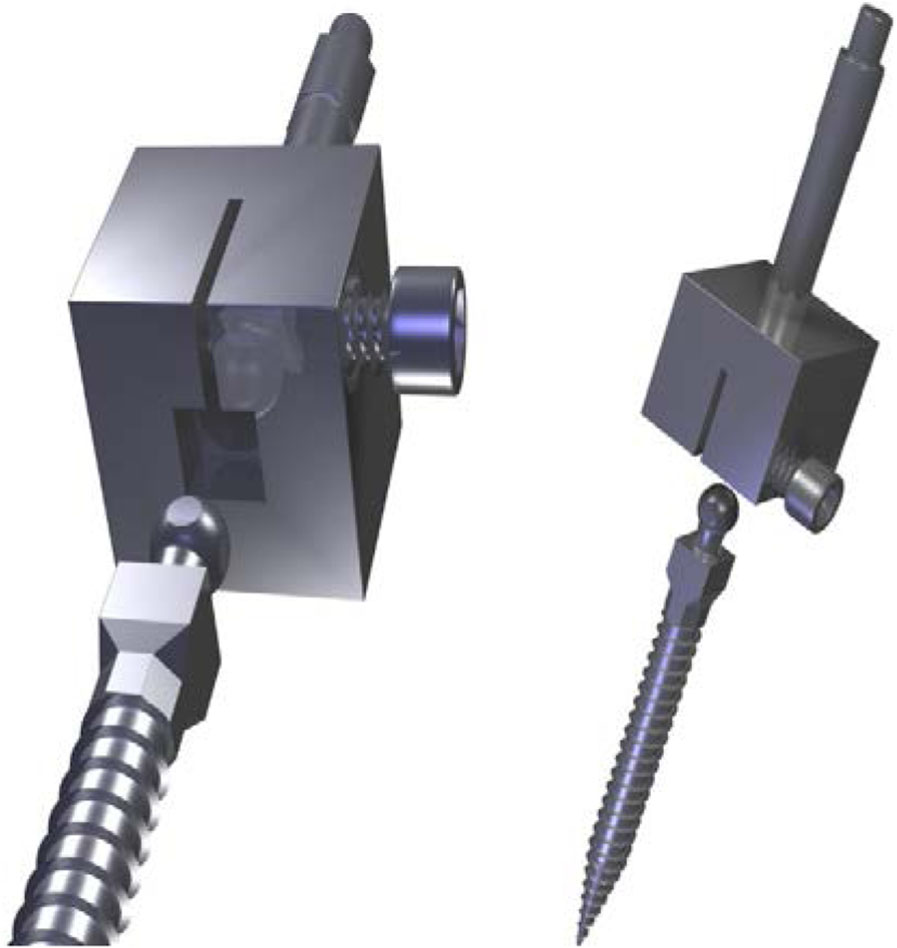
Customized SmartPeg diagrams

Single use Osstell SmartPegs for standard implants are made from a soft metal with a zinc-coated magnet mounted on top of it and attached to the implants or abutments’ internal threads. As the company does not provide SmartPegs for one-piece implants, we developed a customized SmartPeg for mini dental implants (3M™ESPE™ MDIs), which do not have internal threads (Fig. 1). After confirming that the standard SmartPegs™ are fabricated in aluminum, we customized a prototype in the same metal with a square-shaped assembly, which could be tightened with a small screw over the spherical top end of the MDIs. Our SmartPeg prototype was tested for reproducibility verifying the ISQ values on an MDI inserted into a wooden plank made of balsa wood. RFA measurements were taken 50 times, and a standard error of mean of all measurements was calculated.

### Animal model and sample size

Eight clinically healthy New Zealand white rabbits weighing >3.5 kg used for the study were housed in the Central Animal House facility. The head of the tibia/femur of the animals were chosen for the implantation of samples because they have been widely used as an animal model, and so, our results could be promptly compared [39–46]. The sample size of this study has been calculated based on the results of a similar study [36]. It was expected that 88% statistical power would be achieved by using sixteen 3M™ESPE™MDIs (experimental) and equal number of regular implants Ankylos®, Dentsply Friadent GmbH (control). Each animal received two implants on each of the hind limbs, i.e., the right and left tibia/femur heads, randomly. Therefore, each animal had a total of four implants, i.e., two experimental and two regular implants, randomly located.

### Surgical procedures

The procedures were approved by the institutional animals’ ethics review board of McGill University, Montreal, Canada. Adequate measures were taken into consideration to minimize pain and distress in the animal during the procedure. Animals were anesthetized by intravenous injections of a ketamine hydrochloride-xylazine mixture at 35–50 and 1–3 mg/kg, respectively, according to the method described by Green et al. [47]. Acepromazine was injected subcutaneously at the dosage of 1 mg/kg. Further injections of the mixture were given to maintain anesthesia, if necessary. All surgical procedures were performed in accordance with McGill’s standardized operating protocol (SOP).

For the MDIs, a small longitudinal skin incision was made just distal to the tibia/femur joint. The tibia/femur head was exposed subperiosteally, and an osteotomy was performed with the pilot drill under copious irrigation with saline solution, transposing the cortical bone to the depth of 0.5 mm. The implants were aseptically transferred to the bone site and manually rotated clockwise while exerting downwards pressure to start the self-tapping process. When bony resistance was encountered, the winged thumb wrench was used for driving the implant deeper into the bone, if necessary.

Ankylos® implants were inserted in the other tibia/femur head of the animals according to the manufacturer’s protocol as follows: After mobilizing the subperiosteal flap and using a 3-mm center punch to register a guiding point for the osteotomy, a twist drill, depth drill series and a conical reamer were used sequentially to complete the osteotomy and to develop a conical shape for accomodation of the implant’s body. A counterclockwise rotation was used to compress the bone in case of soft bone. The tap or thread cutter was used to create the threads in dense bones. Following, the implant assembly was aseptically transferred to the osteotomy site, and the implant placement was started manually and finalized using a hand ratchet. If excessive force was experienced, the osteotomy was irrigated, and the depth was checked by retapping.

### Resonance frequency assessment

Resonance frequency assessment was performed thrice, just after the insertion of the implants, using the Osstell ISQ™ device. In brief, customized SmartPegs were stabilized onto the head of the 3M™ESPE™ MDIs and Osstell company’s specific SmartPeg™ devices were screwed into Ankylos® implants, taking care to ensure that no significant torquing force was applied to the implants, and the RFA was carried out. These procedures were repeated for post-euthanasia RFA.

### Post-surgical treatment and euthanasia

The rabbits were given a dose of cephalexin 12 mg/kg 0.5 mL IV once intraoperatively and a postoperative analgesic, i.e., carprofen 2–4 mg/kg SC every 8 h for 3 days, according to McGill’s SOP. The animals had a free access to water and food, and routine daily care followed as per McGill’s SOP#524.01. The sutures were removed after 7–10 days, and the animals were euthanized at 6 weeks postoperatively. It has been shown by various authors that this period is adequate to develop a “rigid osseous interface” in rabbits [30]. An overdose of pentobarbital sodium 1 mL/kg intravenously was used for this purpose [48].

### Statistical analyses

ISQ values were averaged and compared between implant types and times using Wilcoxon’s matched pairs signed-rank tests at a significance level of *p* < 0.05. Statistical analysis was performed with the help of SPSS statistical software version 17.

## Results

**Fig. 2:**
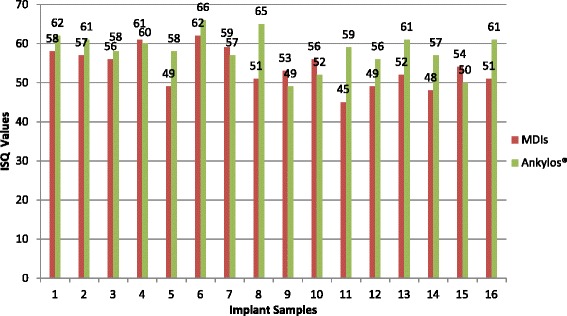
ISQ values of MDIs and Ankylos® immediately upon insertion

**Fig. 3:**
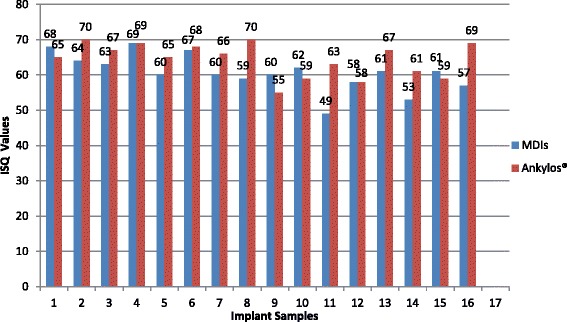
ISQ values of MDIs and Ankylos® after euthanasia

The ISQ values obtained while calibrating the customized SmartPeg were similar to in vivo results. Median ISQ values at insertion and at 6 postoperative weeks were 53.3 (IQR 8.3) and 60.5 (5.5) for the 3M™ESPE™MDIs, and 58.5 (4.75) and 65.5 (9.3) for the Ankylos® implants, respectively, with no statistical difference (Figs. 2 and 3). The ISQ values of both 3M™ESPE™ MDI and Ankylos® (Figs. 2 and 3) increased significantly from the time of insertion to 6-week post-insertion (*p* < 0.05).

## Discussion

It is important to measure the Implant Stability Quotient (ISQ) of single-piece mini dental implants as they are becoming increasingly popular, with the concomitant increase in publications demonstrating their high survival and success rates. Although the clinical use of Osstell devices is also increasing, there is lack of studies on its use with single-piece implants, which do not have internal threads. Implant Stability Quotient (ISQ) is an objective and standardized method for measuring implant stability clinically ranging from 55 to 80, with higher values usually observed in the mandible [49]. The ISQ scale has a non-linear correlation to micro mobility. With more than 700 scientific references, we now know that high stability means >70 ISQ, between 60 and 69 is medium stability, and <60 ISQ is considered as low stability.

The rabbit tibias have been used to determine longitudinal changes in the resonance frequency and measured for over 168 days from the time of implant insertion, and it was observed that resonance frequency values increased over time [38].

However, the relationship between the bone density and ISQ is not significant [50]. Therefore, higher ISQ values are a sign of bone anchorage of implants, but the relationship of resonance frequency analysis with bone structure is unclear [51–53]. ISQ values decline in the first 2 weeks after implant insertion, and these changes may be associated with early bone healing and marginal alveolar bone resorption. Bone remodeling reduces primary bone contact. In the early stage after implant placement, the formation of bony callus and increasing lamellar bone in the cortical bone causes major changes in bone density. Therefore, in the healing process, primary bone contact decreases and secondary bone contact increases [53, 32]. Degidi et al. [54] reported that there may also be a discrepancy as the histological analyses is a two-dimensional picture of the three-dimensional bone-implant contact.

If the initial ISQ value is high, a small drop in stability normally levels out with time. A big drop in stability or decrease should be taken as a warning sign. Lower values are expected to be higher after the healing period. The opposite could be a sign of an unsuccessful implant, and actions should be taken accordingly.

Studies have shown that the resonance frequency value is greatly associated with the quantity of bone-implant contact [31, 38]. There is a positive correlation between resonance frequency analysis and histomorphometric measurements [37]. In our histological study previously reported, similar findings were demonstrated [55].

Our results indicate that both types of implants achieved primary and secondary stability.

Several measurements may be more dependable than single measures; therefore, it may be important to measure resonance frequency multiple times and average the values in order to obtain the most reliable assessment. While reliability of resonance frequency analysis has not been established in the past for these mini dental implants used for overdentures, studies have shown similar or lower levels of reliability for regular dental implants [56].

In general, there was an increase in the ISQ values in both groups, which may be related to enhancement of rigidity between the implants and neighboring tissues and largely with the changes at the bone-implant interface. It has been demonstrated that there is a development of woven bone surrounding the implants 1 week following placement in the rabbit tibia. This scantily organized bone is resorbed by osteoclasts and slowly remodeled into the lamellar bone and gets more compacted around the implant surface and remodeled to become a mature bone over a period of 42 days [38, 57]. There seems to be minimal changes in the resonance frequency after this period. Our results are in concurrence with the study by Meredith et al. [38].

As there are no studies that provide data based on resonance frequency measurements for single-piece MDIs, the exact RFA threshold values for MDIs may have to be identified with more studies conducted in vivo.

The resonance frequency assessment with a customized SmartPeg would be a useful tool to provide clinically useful information about the condition of the bone-implant interface of 3M™ ESPE™MDIs. Frequently, implant failures are associated with biomechanical reasons; implant stability assessment can reduce this to a great extent. The higher the RFA value, the higher the success in implant treatment and the lower the risk for failure in the future. On the other hand, lower RFA values may indicate greater risk for implant complications. The MDIs are usually immediately loaded. Resonance frequency measurement technique is also of value in evaluating the immediate loading implants [58]. The results of the present study are encouraging and show that it is possible to measure ISQ for these single-piece MDIs. This study is the first of its kind and similar type of studies should be conducted among humans, to make the results more meaningful and generalizable.

## Conclusions

The results of this animal study indicate that ISQ measurement of these single-piece MDIs is possible with the help of a custom-made SmartPeg and that 3M™ESPE™MDIs attain primary and secondary stability at the same levels as standard implants in the rabbit tibia.
